# Cardiovascular Comorbidities in Relation to the Functional Status and Vitamin D Levels in Elderly Patients with Dementia

**DOI:** 10.3390/diagnostics12122994

**Published:** 2022-11-30

**Authors:** Violeta Diana Oprea, Mihai Marinescu, Corina Rișcă Popazu, Fabiola Sârbu, Gelu Onose, Aurelia Romila

**Affiliations:** 1 Faculty of Medicine and Pharmacy, “Dunărea de Jos” University, 800216 Galați, Romania; corinapopazu@yahoo.com (C.R.P.); sarbu_fabiola@yahoo.co.uk (F.S.); aurelia.romila@yahoo.com (A.R.); 2 “St. Apostle Andrei” Clinical Emergency County Hospital, 800578 Galați, Romania; 3 “Elisabeta Doamna” Psychiatric Hospital, 800179 Galați, Romania; 4 Department of Physical and Rehabilitation Medicine, Faculty of Medicine, “Carol Davila” University of Medicine and Pharmacy in Bucharest, 020021 Bucharest, Romania; geluonose@gmail.com; 5 “Bagdasar Arseni” Clinical Emergency Hospital, 041915 Bucharest, Romania

**Keywords:** dementia, cognitive decline, cardiovascular risk, functional status, depression, vitamin D deficiency

## Abstract

(1) Background: As dementia is an incurable, multifactorial neurodegenerative disease, we gathered and analyzed a number of patient characteristics, assessing possible correlations that may support early diagnosis and a more accurate prognosis for cognitively impaired patients. (2) Methods: We used standard clinical parameters (cognitive and functional status, comorbidities, and plasma vitamin D levels) in a study group of 162 patients aged above 55 years old. (3) Results: We reported a higher incidence of cardiovascular and metabolic comorbidities in patients with severe or moderate cognitive impairment; a validated correlation between functional status, cognitive status, and serum vitamin D levels; and a more frequently associated profile of neurologic comorbidities in patients with a more significant cognitive deficiency. (4) Conclusions: The present research adds data on the significant correlations of cognitive deficits with cardiovascular, metabolic, and neurologic diseases (and the lack of correlation with osteoarticular illness). Clinicians should make the best use of the current screening and assessment tools (such as the functional scoring of daily activities, cognitive evaluation, and the screening of risk factors). Our data may offer starting points for future in-depth analysis of dementia-modifiable risk factors.

## 1. Introduction

In the context of better healthcare and quality of life, together with an evolving multifactorial etiology and risk factors, the aging population highlights the growing burden of dementia for individuals, families, and society [1,2]. The age-specific incidence rates for dementia were estimated to decrease by approx. 13% per decade starting from 1988 in the developed countries (United States and those in Europe) [2,3,4,5,6]. We have seen increasing periods for both dementia-free life expectancy and years of survival with dementia; nevertheless, epidemiologists have foreseen a growth in the total number of dementia cases [4,5]. Reducing the burden of dementia has been identified as a key global health priority [1,2,6]. As no disease-modifying therapies are yet available, the most significant present strategies address the known modifiable risk factors and integrate different disciplines as a holistic treatment process. Healthcare systems and organizations are urged to consider decreasing the impact of dementia, as it represents a significant concern due to the increasing cost of dementia care and the high family and societal impacts of the disease [4,6,7].

Dementia is an incurable neurodegenerative disease of an unknown cause. The etiology of dementia has been proven to be multifactorial: some aspects are non-modifiable risk factors (such as genetic factors or family history and advanced age) but many can be influenced throughout a lifetime, thereby presenting the possibility of reducing the likelihood of developing dementia or at least delaying its onset [3,5,6]. A review published in 2017 by the Lancet Commission [8] presented compelling evidence on nine potentially modifiable risk factors for dementia: limited education, hypertension, hearing loss/impairment, smoking, obesity, depression, limited physical activity, diabetes mellitus, and reduced social contact. In addition to these, other potentially modifiable risk factors for dementia have been suggested, such as hypercholesterolemia and cardiovascular diseases; additionally, a potential prognostic role of vitamin D deficiency has been proposed [2,7,8,9,10,11,12]. Some divergent research results have been communicated regarding the influence of vitamin D and related risk factors (such as mobility or sun exposure); studies with very different target populations, research hypotheses, and heterogeneous trial designs have not been able to clarify a consensus resolution on the possible correlations linking vitamin D deficiency or its supplementation with evidence of their negative or positive impact for dementia care [8,9,11].

A link between hypovitaminosis D and neurocognition, particularly regarding major neurocognitive disorders, has been demonstrated by numerous recent studies where the pleiotropic effects of vitamin D were described. Testing for seric 25-hydroxy vitamin D was added to other biomarkers recommended by international guidelines for neurocognition screening, such as vitamin B12 deficiency and hypothyroidism in patients with Mild Cognitive Impairment (MCI) or dementia [11,12].

Vascular dementia, accounting for around 20% of all dementia cases, is usually associated with cerebrovascular diseases such as strokes and lacunar infarcts, hemorrhages, cardio-embolism [9,13,14,15], and other comorbidities such as hypertension and diabetes mellitus. Vascular changes (both micro-and macroangiopathic) play an important role in the pathogenesis of vascular dementia [14,15,16]. 

Alzheimer’s Disease (AD), the most common subtype of dementia affecting 60% to 80% of dementia patients, registers vascular dysregulation thought to precede the identification of classical biomarkers associated with AD, although the exact relationship of cerebral blood flow (CBF) alterations with the progression of early AD is still unknown [15,16]. 

We found recently published data on systematic reviews and original research demonstrating that cardiovascular risk factors are associated with cognitive decline and that the longitudinal, cumulative trajectory of cardiovascular risk is predictive of dementia risk, being linked with the emergence of memory decline. There is controversial evidence on hypertension control having an impact on progress toward dementia; this suggests the possibility of a U-shaped relationship between blood pressure and cognition [7,9,15].

The inconsistency of the recent research data and the diversity of trial designs and study populations make the correlations between all these different risk factors difficult to assess. These things considered, we analyzed a single-center geriatric patient lot from the perspective of different possible associations of cognitive decline with some comorbidities, functional status and mobility, and plasma vitamin D levels. 

## 2. Materials and Methods

We conducted a retrospective, non-interventional study on a lot of 162 patients aged 55 years and older who had hospitalized in a geriatric specialty clinic during a period of 24 months (starting January 2020), aiming to assess any possible correlations that may suggest a link between cognitive decline and other factors. The median age was 75.4 years (55–93 years old interval) with a female:male gender ratio of 3:1. The study lot was split based on cognitive function into an active subgroup of 118 cognitively impaired patients and a control subgroup of 44 patients with normal cognitive status.

All consecutive patients admitted to the Geriatric specialty department (either by direct referral from a family physician or through hospital Emergency Room recommendation) were included for the analysis. Patients with acute illness requiring emergency interventions and who were unable to undergo a cognitive assessment were not included in the analysis. In addition, an exclusion criterion was set for patients under therapy with Vitamin D supplements in the previous 12 months prior to hospital presentation, in order to avoid bias in the assessment of any correlations of vitamin D status.

The study protocol included careful registration of comorbidities; the most prevalent ones were subsequently classified into 4 major categories:

- Cardiovascular: hypertension, cardiac failure, coronary heart disease, myocardial infarction, bundle branch blocks, atrial fibrillation, cardiomyopathy, valvular heart diseases, atherosclerosis, deep vein thrombosis, and arteritis;
- Metabolic: diabetes mellitus, dyslipidemia, obesity, and hypercholesterolemia;
- Osteo-articular: osteoarthritis, osteoporosis, arthrosis, rheumatoid arthritis, and Paget’s disease;
- Neurological: sequelae of cerebral stroke, Parkinson Disease, epilepsy, and Huntington’s Disease.

The cognitive evaluation was performed using the following standard testing procedures: the Mini-Mental State Examination (MMSE), Clock-Drawing Test, and Montreal Cognitive Assessment [17,18,19]; associated depression was diagnosed by a psychiatric specialist and was evaluated using the Geriatric Depression Scale (GDS) [10,20,21,22,23]. The patients’ cognitive statuses defined the allocation of cases into two study subgroups: one consisting of 118 patients with cognitive impairment (either mild, moderate, or severe) and another consisting of 44 patients—defined as the control group—with normal cognitive function.

Functional status and mobility were also considered as study parameters; the main instruments included in this hospital admittance assessment were: Activities of Daily Living (ADLs) assessed using the Katz Index of Independence in ADLs [24,25,26,27,28] and Instrumental Activities of Daily Living (IADLs) based on Lawton IADLs Scale [29,30]. These checklists indicated patients’ functional independence of self-care [25,26,27,28,29,30]. The version of the Katz Index involves scoring 6 basic ADLs including bathing, dressing, transfer, toileting, feeding, and continence [24,26], while the Lawton IADLs scale includes 8 activities (the ability to use the phone, shopping, meal preparation, housekeeping, laundry, transportation, the responsibility for administering one’s own medication, and the ability to manage one’s own finances). These two instruments are included in the local Hospital Standard Geriatric Evaluation Protocol, as they are simple, fast, and reliable assessment tools for elder patients.

The serum 25-hydroxy vitamin D (25(OH)D) concentration was measured with rapid indirect competitive fluorescent immunoassay designed for the quantitative measurement of 25-Hydroxy vitamin D and related hydroxylated metabolites in human serum and plasma (K3 EDTA, lithium-heparin, and citrate) specimens. Based on serum 25(OH)D levels, vitamin D deficiency is defined as any value less than 30 ng/mL, with severe deficiency defined as less than 20 ng/mL and insufficiency between 21 and 29.99 ng/mL, and these thresholds were used for interpretation of study findings [12,31,32]. 

Main stratification data used for statistical analysis are shown below (Table 1, Table 2, Table 3 and Table 4).

The statistical methods used to analyze and interpret the results were based on correlation (such as Pearson’s and ANOVA test), hypothesis testing, and regression methods. During the analysis by normality non-parametric tests (One-Sample Chi-Square Test and One-Sample Binomial Test), all null hypotheses for the 9 studied covariates were rejected, which was based on the significance coefficient below target (*p* < 0.05). The statistical analysis of case summary (IBM SPSS Statistics for Windows, version 28 (IBM Corp., Armonk, NY, USA) was utilized for the qualitative assessment.

## 3. Results—Statistical Analysis

The study lot of 162 hospitalized patients included cases admitted to the Geriatric Clinic for complaints linked to one or more categories of symptoms, both related and unrelated to dementia. There were 127 women (78.4%) and 35 men (21.6%) and the median age in the study lot was 75.4 years old. Based on cognitive function, a control group of 44 patients with normal cognition status was considered for evaluation versus an active, cognitively impaired subgroup of 118 patients.

The demographic details and the mean duration of hospitalization are presented below in Table 5.

When we examined all the associated comorbidities, both dementia and non-dementia patients had various diagnoses, most of them with more than five associated comorbidities (90.1% of all patients in the study lot suffered from at least five diseases). Based on ICD-10 criteria, these diagnostics were clustered into four categories: cardiovascular diseases (hypertension, atrial fibrillation, heart failure, coronary disease, status post-myocardial infarction, etc.), metabolic diseases (diabetes mellitus, dyslipidemia, hepatitis, chronic kidney disease, etc.), osteoarticular illnesses (osteoarthritis, rheumatoid arthritis, osteoporosis, etc.) and neurologic conditions (stroke and its sequelae, Parkinson’s disease, epilepsy, etc.), which are all shown in Table 6 and Table 7. 

All the collected data, organized by four subgroups based on each patient’s cognitive status measured by MMSE, were analyzed by normality non-parametric tests and all null hypotheses for the nine studied covariates were rejected based on the significance coefficient below the target (*p* < 0.05).

Correlations were tested with regard to any association versus age groups, the level of cognitive impairment, the level of serum vitamin D, functional status, depression, and the type of comorbidity. The age sub-categories consisted of 1: 55–64 years old, 2: 65–69 years old, 3: 70–74 years old, 4: 75–79 years old, 5: 80–84 years old, and 6: 85–100 years old. The results are presented in Table 8. 

The impact of cardiovascular comorbidities regarding the association with hypovitaminosis D is 43%, with a higher level in the 60–69 age groups. Metabolic comorbidities have an impact of >25% for age groups > 75 years. Although residual, the correlation of depression and vitamin D deficiency is mainly manifested in patients in the 65–69 age group.

The analysis of comorbidities shows that the majority of patients in our study group had associated cardiovascular disorders. The segregate analysis revealed a higher incidence of cardiovascular comorbidities in the patients with severe or moderate cognitive impairment compared with the individuals with normal or mildly impaired cognitive function.

Our statistical analysis spotlights the validated correlation between functional status, cognitive status, and serum vitamin D levels. As seen in Table 8, our analysis provides evidence that with age, the impairment of functional and cognitive statuses is correlated and is strongly influenced by vitamin D deficiency. Using the dynamic mean, we observed a decrease of up to 20% versus the general mean value regarding functional and cognitive statuses in patients 80–84 years old, while the 25(OH)D serum levels were only mildly decreased by 1%. In elderly > 80 years old, the decrease versus the general mean value is 25% for functional and cognitive statuses, with a more significant variation of a 14% lower mean value versus the general one. A significant conclusion from our data analysis highlights the correlation of the magnitude of serum vitamin D deficiency with the patients’ functional and cognitive statuses, starting at 80 years old.

Regarding the functional status, there is a quantified influence of 53% of its change in the presence of the alteration of the cognitive status, especially in patients < 70 years old, but after the age of 70, the degree of dependence of the functional status on cognitive function appears to decrease significantly.

A dependence of 25% is noted between the functional status and the presence of neurological comorbidities in the study group, manifesting predominantly in the age group from 65–75 years, the period in which the peak of serum vitamin D deficiency, as well as the severity of depression, is recorded.

With respect to neurologic disorders, we see significant differences between the subgroups split by cognitive status. A total of 50% of patients with lower MMSE scores (a more significant cognitive deficit) also suffer from neurological disorders, while elderly with a normal cognitive status presented neurologic comorbidities only in 16% of cases. In our study group, neurologic comorbidities are more frequently found in patients with a higher degree of cognitive impairment.

Metabolic comorbidities (such as diabetes mellitus or dyslipidemia) are associated with cognitive deficiency in 60% of patients versus 48% of cases in individuals with normal cognitive function.

The Pearson correlation coefficients emphasize that cardiovascular illnesses are correlated with the level of cognitive impairment: in the control group, cardiovascular morbidity has a coefficient of 39.7% (directly proportional model to cognitive status for cardiovascular comorbidities and functional status; depression is inversely proportional to the severity of cognitive deficiency, suggesting that severe depression is more likely to be correlated with MCI).

Using the statistical regression model based on the OLS method (Ordinary Least Squares) for the two subgroups with and without cognitive impairment, based on high statistical significance, we were able to validate the hypothesis that cognitive status correlates with a set of comorbidities (cardiovascular, neurologic, metabolic, and osteoarticular) as well as functional status and depression in as high as 99% of the control group (normal cognition) and 93% in the cognitively impaired subgroup.

In the test model, the Pearson correlation coefficient is significantly different between the two subgroups, with a median Pearson correlation between the regression variables and dependent variables in the control group and a high dependence for some comorbidities (especially cardiovascular) and functional status in the cognitively impaired subgroup. For both sub-lots, we see a valid correlation of the model based on the cognitive function evaluation (Table 9 and Table 10). 

**Figure 1:**
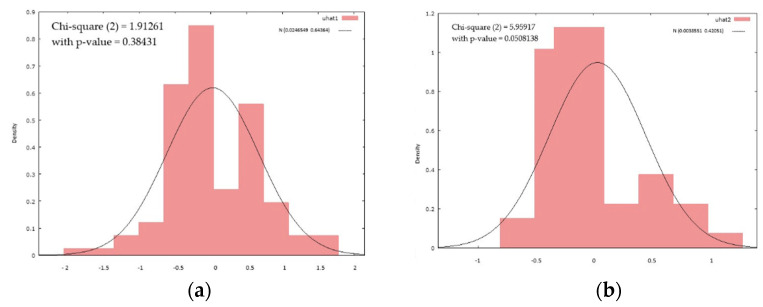
Histograms of Pearson model correlation of cognitive status in the (**a**) active group and (**b**) control group, showing a median Pearson correlation between the regression variables and dependent variables in the control group and a high dependence for some comorbidities (especially cardiovascular) and functional status in the cognitively impaired subgroup.

The graphical distribution shows a higher homogeneity of the models for the subgroup of cognitively impaired patients (Figure 1).

## 4. Discussion

The multifactorial pathogenesis of dementia includes both widely accepted hypothesis, such as the link with cardiovascular diseases or the reduced mobility of the elderly, and other more controversial concepts, such as the deterministic role of vitamin D deficiency in cognitive decline [7,8,11,13,14,15]. Currently, we have limited data regarding the correlation of multiple parameters applied to the same patient cohort; this is the motivation for our research, in which we try to assess the connections between cognitive and functional statuses in patients with different comorbidities and evaluate their possible relation to vitamin D deficiency.

As presented in some previous studies, cardiovascular diseases were highly correlated with the level of cognitive impairment in our active patient group. To a lesser extent, we noted that patients with a more significant functional dependency were more likely to be associated with proportionally correlated cognitive alterations. Our results showed a higher prevalence of cardiovascular comorbidities in the patients with severe or moderate cognitive impairment compared to individuals with normal or mildly impaired cognitive function. This correlation is consistent with most available data; furthermore, we have located systematic reviews confirming the positive impact of cardiac rehabilitation on global cognition in older patients, reporting therein the significant cognitive improvement of the attention/executive function and memory domains [15,32,33,34,35,36].

Cardiovascular comorbidities have been proven to be highly correlated with pathological changes in cerebral circulation, with some researchers emphasizing a common factor, namely, central arterial stiffening [37,38], with up to 50% of patients diagnosed with heart failure having an associated cognitive dysfunction. Although some compensatory mechanisms were described to explain cerebral blood flow autoregulation, heart dysfunction results in impaired perfusion, metabolic insufficiency, and regional or global structural deterioration in the brain. Moreover, recent research showed that the vascular bed of the external carotid artery is an important buffering mechanism for preventing over- or hypo-perfusion in the intracranial cerebral anterior artery due to certain cardiovascular conditions [37,39,40]. The circle of Willis is the primary collateral system protecting the brain from ischemia when occlusions occur in the intracranial arteries, but the arteries in the circle of Willis system are susceptible to atherosclerosis and aneurysms, which highly influence its hemodynamics [39,40].

Interventions addressing cerebral hypoperfusion have been evaluated in some trials regarding AD; improvements in CBF following various interventions (both pharmacological and non-pharmacological) were seen in the early stages of the disease and seem to be correlated with cognitive functioning [25,34,36,41,42,43,44,45,46,47]. Pharmacological therapies may be more beneficial for AD patients if they target mechanisms that become abnormal at the very beginning of the course of the disease when symptoms are minimal. A great challenge would be to identify standard practical biomarkers that can detect these early changes—biomarkers that could become available for current clinical practice and wider use [43,44,48,49,50,51,52,53].

Some recently published evidence focuses on relevant biomarkers related to AD and cardiovascular parameters, proving that poor vascular health does not significantly impact amyloid deposition but may have a direct and indirect effect on AD-related neurodegeneration [43,44,54,55,56,57,58,59]. Aging acts through a number of biological mechanisms at the cellular or tissue level, which may lead to a multi-system loss of function and reserves. The major issue that may have contributed to the controversy could be the confounding effect of age on both vascular health and AD biomarker variables.

Recent research showed evidence for common underlying pathophysiological links between cardiovascular disease and Parkinson’s Disease in the areas of glucose metabolism, cellular stress, lipid metabolism, and inflammation [38,40,56,57,58]. Cardiovascular comorbidities are closely associated with pathological changes in cerebral circulation and relevant neurological symptoms; a recent study showed that components of peripheral blood leukocytes reflect some clinical symptoms of Parkinson’s Disease. Moreover, neuroimaging studies showed that the neutrophil–lymphocyte ratio was correlated with the loss of dopaminergic uptake and connectivity of white matter tracts in certain brain regions, indicating the involvement of peripheral inflammation in the development of neurodegeneration [56,57,58].

We have data demonstrating that lipids play an important role in the pathogenesis of processes upstream of neurodegeneration in dementia, basing our hypothesis on the fact that metabolic comorbidities (especially hypercholesterolemia) could be instrumental to the evolution of cognitive impairment. In this present study, we noted that at least one metabolic disease is associated in 60% of patients with cognitive deficiency versus a weaker direct relationship in individuals with normal cognitive function (48% of cases). Vascular health lowers the threshold for dementia by independently impacting neurodegeneration [36,41]. The vascular systems of the brain and the eyes have some structural and functional similarities, and the connection between AD and the retinal vascular system has been confirmed in several recent studies. This triggered the interest for exploring the use of another ocular biomarker in AD, namely, measuring degenerative processes in the retina and the visualization of the macular choroid, which may support an earlier and non-invasive test for AD diagnosis [36,41,42,43].

Functional status has been demonstrated to be related to cognition; however, the research findings appear mixed, as a true correlation with a causal relationship is difficult to demonstrate [46,47,48,49,50]. Different levels of cognition and/or dementia subtypes may affect the relationship between the impairment of some cognitive domains and functional status. We have evidence that a decline in judgment/problem solving, which is reflective of a decline in cognition, precedes a decline in functional status. Declining functional status and judgment/problem solving among individuals not yet diagnosed with dementia may be an indicator that these individuals need further evaluation of their neurological and cognitive functions.

When evaluating the association of cognitive impairment status within the categories of plasma deficiency of 25(OH)D in our study cohort, the measurements showed that severe mental deterioration is associated with a significant vitamin D reduction versus normal serum levels, whereas the highest percentage of patients with normal 25(OH)D plasma levels belong to the category of normal cognitive function individuals.

Indirect scientific evidence for this correlation was tested in different study designs when investigating the effects of vitamin D supplementation on cognitive outcomes in elderly individuals. There is no clear notion of when vitamin D (natural or administered as a supplement) is most effective in the pathogenesis of cognitive decline, particularly in AD; the pre-existing neurological insult could have been the reason for the failure of introducing such therapy too late or in insufficient doses [32,51,54,59]. Overall, conflicting evidence is provided by studies, which found that vitamin D supplementation neither improved cognitive outcomes nor reduced the risk of dementia or MCI compared to the controls. On the contrary, other research found that those who received oral vitamin D3 supplementation experienced improved global cognition and executive-functioning abilities over a longer follow-up period compared to the controls [55,59].

Our statistical analysis proved that there is a correlation between cognitive and functional statuses with serum levels of 25(OH)D, which increases with age, particularly in much older patients (>80 years old). Recent studies demonstrate the neurosteroid actions of vitamin D in the regulation of calcium homeostasis, its antioxidant and anti-inflammatory properties, and the role in cerebral β-amyloid deposition, as well as the neuroprotective action of vitamin D against neurodegenerative process associated with Alzheimer’s disease and cognition. Animal studies provide evidence on the behavioral and anatomical changes in the hippocampus in animals with low vitamin D levels. We have data that depression may be caused by an imbalance between excitatory and inhibitory pathways in the brain, with a hypothesis arguing that vitamin D reduces the increase in neuronal levels of calcium (CA +2) that drive depression. Vitamin D plays a role in maintaining the expression of the CA 2+ pumps and buffers, specifically, in reducing CA 2+ levels, which may explain how it may influence the onset of depression [54,55,56].

The data we present have some limitations, stemming from the small patient lot size and the perspective of a single geriatric center, but our study explores possible correlations between different parameters in patients diagnosed with dementia. Accounting for cognitive and functional conditions, comorbidities, and vitamin D deficiency, this all-in-one analysis may offer starting hypotheses for further research, bringing supplementary evidence to clarify some current debates in the literature. Applying composite criteria when evaluating the prognostics for dementia patients would probably be the best approach for clinicians. The assessment of patients’ functional status and vitamin D levels—especially in the early stages of dementia—may be a valuable, rapid, and affordable screening tool.

## 5. Conclusions

The present study aims to add further data to the many systematic reviews and meta-analyses of cross-sectional studies, case-control studies, and observational prospective studies that have suggested an association between dementia and low vitamin D levels, cognitive impairment, functional status, and different comorbidities—most of which have received separate consideration. We presented evidence for a strong correlation between cardiovascular diseases and functional dependence with cognitive decline in a study group of dementia patients. In addition, in our study group, vitamin D deficiency strongly influenced the impairment of functional and cognitive status, especially in older patients. The limitations of our work are related to a smaller sample size and the perspective of a single center, so all hypotheses should be further analyzed in larger and more complex cohorts, especially with respect to the complex correlation of cardiovascular comorbidities with functional status and vitamin D deficiency in patients diagnosed with dementia. 

While new, valuable biomarkers are emerging, until these are made available in broad clinical practice, we still need to examine the current toolbox already used in daily routines and corroborate relevant data gathered from complex screening across all neurodegenerative disorders. 

## Figures and Tables

**Table 1:** Assessment criteria of functional status based on ADL/IADL score (maximum score on Katz Index ADL—6; maximum Lawton IADL score—8).

Functional Status Based on ADL/IADL Scales	
Minimal/No functional dependence	ADL: (5–6)/6 and IADL: (7–8)/8
Medium functional dependence	ADL: (3–4)/6 and IADL: (4–6)/8
Important functional dependence	ADL: (0–2)/6 and IADL: (0–3)/8

**Table 2:** Assessment criteria of cognitive status based on MMSE score (range 0–30).

Cognitive Status Based on MMSE Scale	
Normal	24/30–30/30
Mild impairment	19/30–23/30
Moderate impairment	10/30–18/30
Severe impairment	0/30–9/30

**Table 3:** Assessment criteria of cognitive status based on GDS score (range 0–30).

Depression Status Based on GDS Score	
Normal	24/30–30/30
Mild clinical depression	19/30–23/30
Moderate clinical depression	10/30–18/30
Severe clinical depression	≤9/30

**Table 4:** Assessment criteria of vitamin D status based on serum 25(OH)D levels.

Vitamin D Status Based on Serum 25(OH)D Levels (ng/mL)	
Normal	24–30
Mild impairment	19–23
Moderate impairment	10–18
Severe impairment	≤9

**Table 5:** Demographic data of the study lot and the two subgroups, defined by the cognitive status of patients (an active group of 118 dementia patients and a control group of 44 cognitively normal patients).

	Entire LOT (162 Patients)	Active Subgroup (118 Patients)	Control Subgroup 44 Patients)
Median age (years)	75.5 years (55–93)	72.9 years (55–90)	75.4 years (55–90)
Gender—female N (%)	128 (79%)	91 (77.1%)	37 (84.1%)
Gender—male N (%)	34 (21%)	27 (22.9%)	7 (16.9%)
Living area—rural N (%)	53 (32.7%)	36 (30.5%)	17 (38.6%)
Living area—urban N (%)	109 (67.3%)	82 (69.5%)	27 (61.4%)
Education—secondary/high school equivalent N (%)	100 (61.7%)	79 (67%)	21 (47.7%)
Education—university/master’s degree equivalent N (%)	62 (38.3%)	39 (33%)	23 (52.3%)
Median hospital stay (days)	10.5 (2–30 days)	10.6 (2–28 days)	10.4 (2–30 days)

**Table 6:** The number of comorbidities in the study group and the two research subgroups (the active group of 118 dementia patients and the control group of 44 cognitively normal patients) show a high share of patients having ≥5 comorbidities.

	Entire LOT (162 Patients) N (%)	Active Subgroup (118 Patients) N (%)	Control Subgroup (44 Patients) N (%)
More than 5 associated comorbidities	146 (90.1%)	109 (92.3%)	37 (84.1%)
3–4 comorbidities	14 (8.6%)	8 (6.8%)	6 (13.6%)
1–2 comorbidities	2 (63.5%)	1 (62.7%)	1 (65.9%)

**Table 7:** Analysis of the 4 most frequent disease categories in the study group and the two research subgroups (the active group of 118 dementia patients and the control group of 44 cognitively normal patients) indicate an important prevalence of cardiovascular comorbidities in more than 90% of patients.

	Entire LOT (162 Patients) N (%)	Active Subgroup (118 Patients) N (%)	Control Subgroup (44 Patients) N (%)
Cardiovascular comorbidities	152 (93.8%)	113 (95.7%)	39 (88.6%)
Metabolic comorbidities	96 (59.2%)	73 (61.5%)	23 (52.2%)
Osteo-articular comorbidities	103 (63.5%)	74 (62.7%)	29 (65.9%)
Neurologic comorbidities	51 (32.4%)	44 (37.3%)	7 (15.9%)

**Table 8:** Correlation analysis of research parameters based on age sub-groups.

AGE	Cardiovascular Comorbidities (Option Mean 1–2)	Neurologic Comorbidities (Option Mean 1–2)	Metabolic Comorbidities (Option Mean 1–2)	Osteoarticular Comorbidities (Option Mean 1–2)	Functional Status (Option Mean 1–3)	Cognitive Status (Option Mean 1–4)	Depression Status (Option Mean 1–3)	Serum 25(OH)D Status (Option Mean 1–3)
55–64 years old—1	1.14	1.57	1.29	1.71	2.57	3.57	2.57	1.86
65–69 years old—2	1.21	1.68	1.32	1.26	2.47	3.53	2.32	1.53
70–74 years old—3	1.02	1.77	1.37	1.40	2.53	2.98	2.40	1.81
75–79 years old—4	1.00	1.72	1.47	1.19	2.25	2.91	2.22	1.81
80–84 years old—5	1.03	1.64	1.42	1.36	1.97	2.24	2.24	1.70
85–100 years old—6	1.05	1.62	1.52	1.43	1.67	2.14	2.33	1.48
	1.06	1.69	1.41	1.36	2.25	2.82	2.33	1.72

**Table 9:** Pearson correlation coefficient in the Model ACTIVE GROUP: OLS (Dependent variable: COGNITIVE STATUS) shows a high dependence for some comorbidities (especially cardiovascular) and functional status in dementia patients (S.D. = standard deviation; S.E. = standard error).

	Coefficient	Std. Error	t-Ratio	*p*-Value
Cardiovascular comorbidities	0.917779	0.240415	3.817	0.0002
Neurologic comorbidities	0.135351	0.120395	1.124	0.2633
Metabolic comorbidities	0.113093	0.114933	0.9840	0.3272
Osteoarticular comorbidities	0.0309263	0.121131	0.2553	0.7989
Functional status	0.727940	0.0739723	9.841	<0.0001
Depression status	−0.222393	0.0963808	−2.307	0.0229
Mean dependent variable	2,381,356		S.D. dependent variable	0.846506
Residual sum of squares	4,648,186		S.E. of regression	0.644218
Uncentered R-squared	0.938271		Centered R-squared	0.445582
F (6, 112)	2,837,309		*p*-value (F)	2.70 × 10^−65^
Log-likelihood	−112.4690		Akaike criterion	2,369,380
Schwarz criterion	2,535,622		Hannan–Quinn criterion	2,436,879

**Table 10:** Pearson coefficient correlation in the Model CONTROL GROUP: OLS (Dependent variable: COGNITIVE STATUS) shows a lack of correlation between comorbidities, functional status, and depression in the control group (S.D.= standard deviation; S.E.= standard error).

	Coefficient	Std. Error	t-Ratio	*p*-Value
Cardiovascular comorbidities	0.396548	0.231532	1.713	0.0949
Neurologic comorbidities	0.407316	0.172687	2.359	0.0236
Metabolic comorbidities	0.322223	0.124036	2.598	0.0133
Osteoarticular comorbidities	0.322766	0.129443	2.493	0.0171
Functional status	0.398653	0.117984	3.379	0.0017
depression status	0.296002	0.108564	2.727	0.0096
Mean dependent variable	4,000,000		S.D. dependent variable	0.000000
Residual sum of squares	6,785,023		S.E. of regression	0.422556
Uncentered R-squared	0.990362		Centered R-squared	NA
F (6, 38)	6,508,003		*p*-value (F)	1.02 × 10^−36^
Log-likelihood	−21.30491		Akaike criterion	5,460,982
Schwarz criterion	6,531,496		Hannan-Quinn criterion	5,857,980

## Data Availability

The data presented in this study are available on request from the corresponding author. The data are not publicly available due to local regulations regarding doctoral research.
